# Surgical management of pancreaticopleural fistula with video-assisted retroperitoneal pancreatic debridement: A case report

**DOI:** 10.1016/j.ijscr.2019.10.068

**Published:** 2019-11-06

**Authors:** María Laura Daza Fernández, Liliana Cuevas López

**Affiliations:** a Department of General Surgery, San Ignacio University Hospital, Faculty of Medicine, Pontificia Universidad Javeriana, Bogotá, Colombia; b Department of General Surgery, San Ignacio University Hospital, Faculty of Medicine, Pontificia Universidad Javeriana, Bogota, Colombia

**Keywords:** Video-assisted retroperitoneal necrosectomy, Pancreaticopleural fistula, Pancreatic necrosis, Case report

## Abstract

**Introduction:**

Pancreaticopleural fistula is a rare complication of both acute and chronic pancreatitis. Here, we present a case of a pancreaticopleural fistula treated with Video-Assisted Retroperitoneal Debridement (VARD).

**Case presentation:**

A 52-year-old male with a history of severe acute biliary pancreatitis presented with cough, chest pain and fever. Physical exam showed absent respiratory sounds in the right base. Chest tomography revealed a right pleural collection. The patient underwent a right thoracotomy which confirmed a pancreaticopleural fistula, and then an ERCP was performed for conservative management and the patient was discharged. The patient was re-admitted on the 27^th^ postoperative day where we confirmed a superinfected pancreatic necrosis and persistence of the fistula; the patient underwent a VARD for the treatment of both conditions. The patient was discharged after completing 5 weeks of antibiotic management.

**Discussion:**

The management of a pancreaticopleural fistula is based on medical and endoscopic treatment; the surgical options are reserve for cases where the conservative management have failed and when the spontaneous closure of the fistula is unlikely. In our case, conservative management for the pancreaticopleural fistula failed and the patient presented another complication of the pancreatitis, for which we decided to perform a minimally invasive resective surgery to treat both complications.

**Conclusion:**

This is the first case of a pancreaticopleural fistula with superinfected pancreatic necrosis managed with a video-assisted retroperitoneal pancreatic necrosectomy. This case will give surgeons another tool to treat these conditions that has less complications than other resective pancreatic surgeries and can solve both complications.

## Highlights

• Pancreaticopleural fistula is a rare complication of pancreatitis.
• Surgical managemente is reserve for cases where the conservative management have failed.
• Video-assisted retroperitoneal pancreatic necrosectomy can be a minimally invasive surgical treatment for a pancreaticopleural fistula.

## 1 Introduction

Pancreaticopleural fistula occurs in 0.4% of patients with pancreatitis, it is associated with alcoholic pancreatitis in 67% of cases, and as a complication of chronic pancreatitis in 70%–90% of cases [1,2]. This entity occurs as a consequence of a disruption in the pancreatic duct, whose amylase rich secretion with highly lytic characteristics dissects through the fascial planes and forms a posterior retroperitoneal collection, which then ascends, generally through the hiatus, and dissects the pleural cavity [1,3]. The most common symptoms are dyspnea, chest pain, cough and abdominal pain, which implies a challenge and possible delay in diagnosis [1,4]. The pleural effusion associated with pancreaticopleural fistula is typically refractory to drainage and tends to accumulate rapidly [1]. The most important diagnostic procedure is thoracentesis for measurement of amylase in pleural fluid, followed by imaging studies to visualize the fistulous tract [4]. The usual management is based on three lines of treatment: the first is the conservative management which includes suspension of the oral intake, initiation of total parenteral nutrition and administration of somatostatin analogues; the second line is based on endoscopic management by placing a stent in the pancreatic duct, and the third line is the surgical option, consisting of resective surgery (distal pancreatectomy) and enteric shunts (pancreatojejunostomy, cystojejunostomy) [5]; there are no reports of minimally invasive surgical management as the case we present below, which was definitively managed through video-assisted retroperitoneal pancreatic necrosectomy on an academic practice hospital.

The work has been reported in line with the SCARE criteria [6].

## 2 Case presentation

**Fig. 1:**
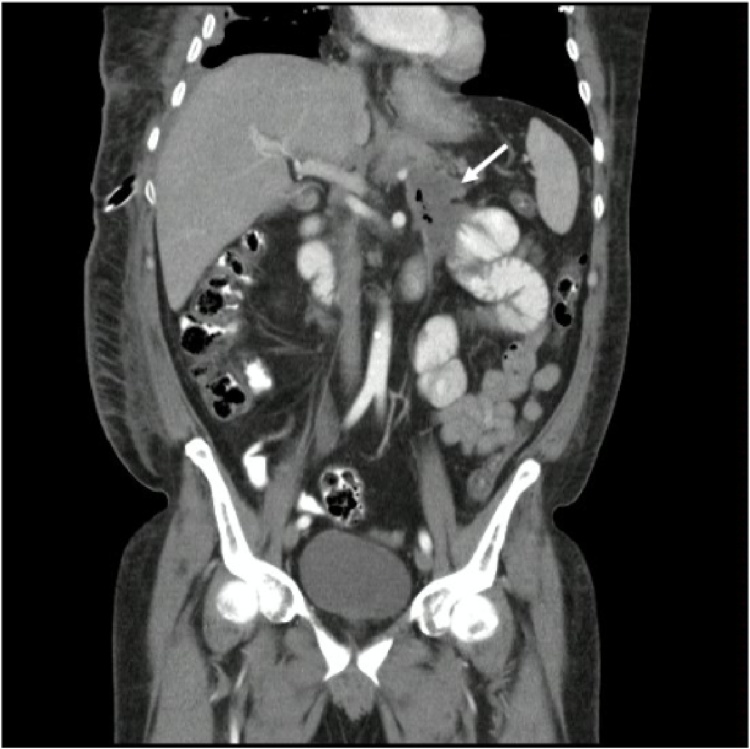
Double contrast Computed Tomography of the abdomen. Coronal reconstruction. The white arrow indicates the peripancreatic collection towards the tail of the pancreas.

**Fig. 2:**
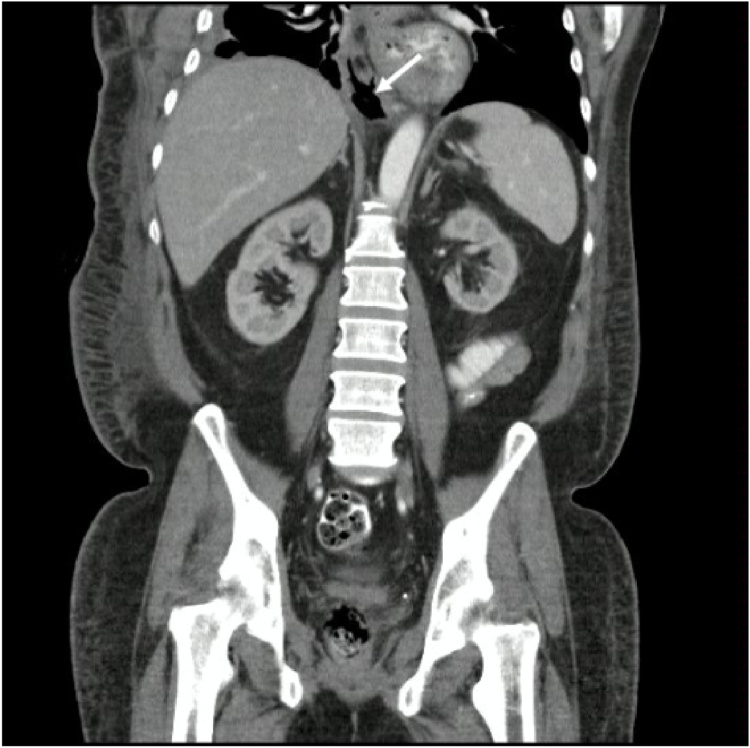
Double contrast Computed Tomography of the abdomen. Coronal reconstruction. The white arrow indicates communication through the diaphragmatic hiatus between the right pleural space and the peripancreatic collections.

A 52-year-old merchant, with a history of an episode of severe acute pancreatitis of biliary origin 2 months before admission, consulted for 3 weeks of cough, chest pain in the right hemithorax and fever, he had no family history of illness, drugs or alcohol consumption. Physical examination revealed absent respiratory sounds in the right base and fine rales in both lung fields. Chest tomography revealed a right pleural collection with a hydro-aerial level of 16 × 11 × 10 cm associated with passive atelectasis of the lower lobe, communicated through the diaphragmatic hiatus with peripancreatic collections in the head and tail of the pancreas (Fig. 1, Fig. 2).

The patient was taken to right posterolateral thoracotomy due to suspicion of pulmonary abscess by a senior Thoracic Surgeon and a third year General Surgery resident, finding a pleural collection of 300 cc, disruption in the lung parenchyma in the posterior basal segment of approximately 1 × 1 cm and disruption in the posterior costodiaphragmatic recess of 1 cm with evidence of pancreatic tissue. An anterior and a posterior chest tubes were left in the chest cavity; postoperative pain was managed with thoracic epidural blockade by the anesthesiologist with adequate pain control. The amylase level in the pleural fluid was greater than 24,000 I/U, confirming the suspicion of a pancreaticopleural fistula, and the pathology report of the tissue sample taken in surgery showed steatonecrosis without the presence of viable pancreatic tissue.

An endoscopic retrograde cholangiopancreatography (ERCP) was carried out for medical management of the fistula, finding a disruption of the distal portion of the main pancreatic duct with extravasation of the contrast medium, a papillotomy was performed, and a 5 cm × 5 Fr pancreatic plastic stent was placed. The patient was put on treatment with octreotide, but it was stopped on the third day due to adverse effects (nausea, vomiting and headache). As the patient presented progressive decrease of drainages volumes through closed thoracostomy tubes, the anterior chest tube was removed, and he was discharged to home care in a temporary shelter with the posterior tube due to the long-distance living place of the patient from a medical center

The patient was re-admitted on the 27th postoperative day due to abdominal pain and purulent drainage through the chest tube. A superinfected pancreatic necrosis was suspected, and the patient underwent a CT-guided percutaneous drainage with placement of a retroperitoneal pig-tail drain, isolating a multi-sensitive Enterococus faecalis in the culture obtained of the pancreatic necrosis. A new ERCP was done without finding the pancreatic stent or disruption of the main pancreatic duct, but there was an extravasation of the contrast medium in the distal portion of the tail of the pancreas, it was decided not to place a new stent given the distal location of the fistula with the permeable pancreatic duct.

**Fig. 3:**
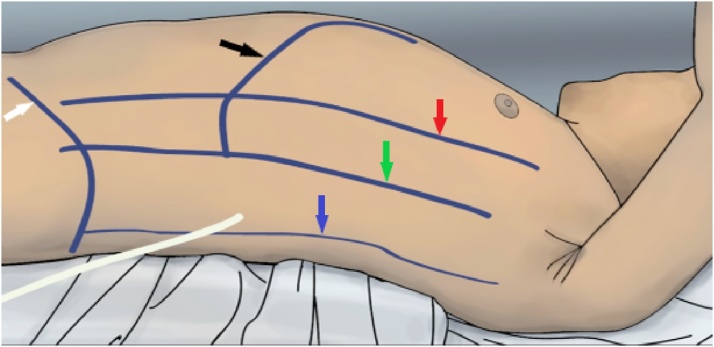
Anatomical landmarks. Black arrow: left twelfth rib. White arrow: anterior superior iliac spine. Red arrow: anterior axillary line.

Given the superinfection of the pancreatic necrosis and the persistence of the pancreaticopleural fistula, the attending junior General Surgeon and a third year General Surgery resident decided to perform a second surgery to manage bothcomplications of pancreatitis, choosing a resective and minimally invasive surgery: a left video-assisted retroperitoneal pancreatic necrosectomy. The patient was in anti-Trendelemburg position with de wrist rotate 30° to the right, the anatomical landmarks were marked to help delimiting between the abdominal cavity and the retroperitoneal space (Fig. 3).

**Fig. 4:**
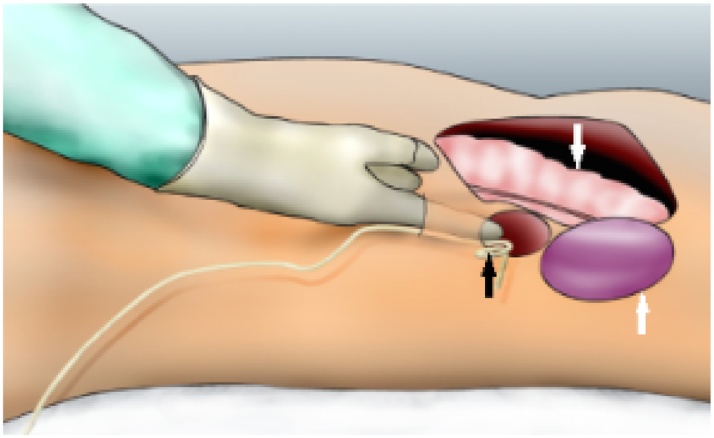
Digital dissection to enter the retroperitoneal cavity and locate the pig-tail drain (black arrow) into the superinfected necrosis. Bellow the walled-off-necrosis is locate the peritoneal cavity (white arrows: colon and spleen).

Then, a 5 cm transverse incision between the anterior and the mild axillary lines was done, 2 cm below the twelfth left rib; then the subcutaneous tissue was dissected, and the muscular plane of the abdominal wall was cut separately, first the external oblique, second the internal oblique, and finally the transversus abdominis was cut carefully, so as not to enter the peritoneal cavity inadvertently. Next, a tunnel was created with digital dissection to enter the retroperitoneal cavity was done; the retroperitoneal pig-tail drain was identified, and it served as a guide to locate the superinfected necrosis (Fig. 4).

**Fig. 5:**
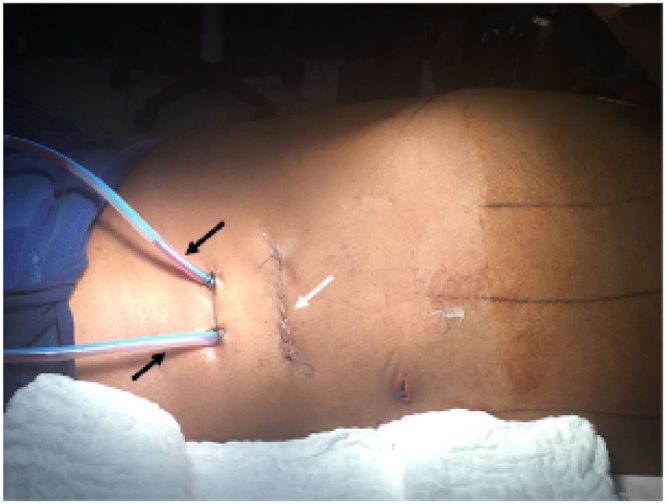
Position of the patient at the end of the procedure. Surgical incision (white arrow) and closed drains left in the pancreatic bed (black arrows) are shown.

**Fig. 6:**
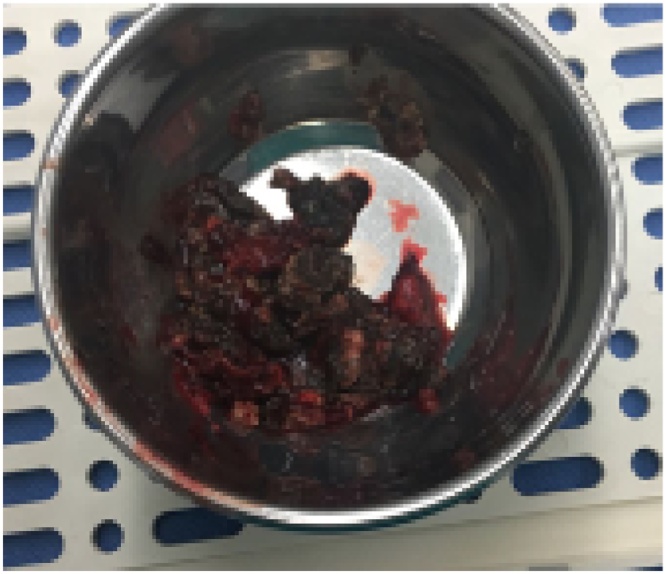
Necrotic pancreatic tissue sample obtained in VARD.

The tunnel was dilatated and a 12 mm trocar was introduced and the carbon dioxide insufflated up to 15 mmHg, a 30° laparoscopic lens was introduced finding necrotic pancreatic tissue with scarce purulent collection around it, debridement was done by blunt dissection with forester forceps and irrigation; two closed drains (Johnson & Johnson Blake drain) of 19 Fr were left in the pancreatic cell (Fig. 5, Fig. 6)

The patient presented an adequate postoperative evolution without fever, pain or oral intake intolerance. An abdominal tomography was performed 3 weeks after the necrosectomy with complete resolution of the peripancreatic collections and the pancreaticopleural fistula, so the right chest tube and the Blake drains were removed. A laparoscopic cholecystectomy was performed by the attending junior general surgeon 3 weeks after the pancreatic necrosectomy without complications and 5 weeks of antibiotic treatment with ampicillin-sulbactam were completed due to superinfection of pancreatic necrosis. Patient follow-up was done by telephone to ensure adherence because of transportation difficulties for the patient, at 1 month, 3 months, 6 months and 1 year, without evidencing any complication.

The patient was satisfied with the definitive surgical treatment for both pathologies since he has not required additional trips to health care centers and has been able to continue with his work and daily activities without limitations.

## 3 Discussion

The management of pancreaticopleural fistula has been described in case reports and case series, but there are no randomized studies given its low incidence [4]. Successful medical management can offer between 30% and 60% of resolution of the fistula, with a 15% recurrence, and a period between 2–3 weeks is given for conservative management [2]. Endoscopic therapy based on placing a stent in the pancreatic duct is used to restore its continuity; it is used in cases of disruption of the pancreatic duct in the body or tail and distal stenosis to the disruption site, with a success rate close to 100% [2]. Surgical management is reserved for patients in which medical and endoscopic management has failed, and for those in whom it is thought that the possibility of spontaneous closure of the fistula is very low; surgical management consists of pancreatic resection and according to the case, pancreatoenteric anastomosis to achieve an adequate drainage of pancreatic secretions [1,4].

In our case, conservative management with somatostatin analogues was stopped due to intolerance, and endoscopic management by placing a stent in the pancreatic duct for 3 weeks did not resolve the fistula. Additionally, the patient presented a second complication, a superinfection of the pancreatic necrosis, for which we decided to perform a minimally invasive resective surgery to treat both complications. With video-assisted retroperitoneal pancreatic necrosectomy, about which we have not found descriptions for this condition, the resolution of both complications was achieved: pancreatic necrosis and pancreaticopleural fistula.

## 4 Conclusion

We present a case of pancreaticopleural fistula in a 52-year-old man. The patient underwent a right thoracotomy that confirmed the fistula, and then an ERCP was performed for a conservative treatment, on the 27th postoperative day we confirmed an infected pancreatic necrosis and the persistence of the fistula, so the patient underwent a VARD for the treatment of both conditions. The current literature shows that the management of a pancreaticopleural fistula is based on medical and endoscopic treatment; surgical options are reserved for cases in which conservative management has failed and when spontaneous closure of the fistula is unlikely. In our case, the conservative management of the pancreaticopleural fistula failed and the patient developed a second complication of the acute pancreatitis, so we decided to perform a minimally invasive resection surgery not previously described in the literature to treat both complications obtaining favorable results.

## Sources of funding

This research did not receive any specific grant from funding agencies in the public, commercial, or not-for-profit sectors.

## Ethical approval

This study design and manuscript were approved by the Pontificia Universidad Javeriana and Hospital Universitario San Ignacio Ethics and Research Committee on May of 2018. All patient data and photographs were de-identified.

## Consent

Informed consent was obtained from the patient for publication of this case report and accompanying images.

## Author’s contribution

María Laura Daza Fernández: Data Acquisition, Data Interpret and writing of the manuscript.

Liliana Cuevas López: Paper Conception, supervised the work, give final approval of the version to be submitted.

## Registration of research studies

Research Registry Unique Identifying Number: researchregistry5194.

## Guarantor

María Laura Daza Fernández, Liliana Cuevas López.

## Provenance and peer review

Not commissioned, externally peer reviewed.

## Declaration of Competing Interest

No potential conflict of interest relevant to this article was reported.
